# Die globale Masernkrise – Ursachenvielfalt von bewaffneten Konflikten bis Impfskepsis

**DOI:** 10.1007/s00103-020-03241-5

**Published:** 2020-11-13

**Authors:** Luisa Denkel, Werner Espelage, Dorothea Matysiak-Klose, Thomas Morwinsky, Anette Siedler, Sandra Beermann

**Affiliations:** 1 Abteilung für Infektionsepidemiologie, FG33 – Fachgebiet für Impfprävention, Robert Koch-Institut, Seestraße 10, 10315 Berlin, Deutschland; 2 Zentrum für internationalen Gesundheitsschutz, ZIG1 – Informationsstelle für Internationalen Gesundheitsschutz, Robert Koch-Institut, Berlin, Deutschland; 3 Kommando Sanitätsdienst der Bundeswehr, München, Deutschland

**Keywords:** Masern, Internationale Ausbrüche, Globale Zunahme, Bekämpfungsbarrieren, Measles, International outbreaks, Global increase, Elimination barriers

## Abstract

**Hintergrund und Ziel:**

Obwohl seit vielen Jahren ein sicherer und effektiver Impfstoff verfügbar ist, nehmen Fälle von Masern seit 2018 weltweit wieder zu. Ziel dieses Berichts ist die Identifizierung möglicher Gründe für diese Entwicklung.

**Methode:**

Es erfolgte eine selektive Literaturrecherche sowie die Auswertung aktueller Berichte und Daten der Weltgesundheitsorganisation (WHO), des Kinderhilfswerkes der Vereinten Nationen (UNICEF) und der Weltbank.

**Ergebnisse:**

Madagaskar, die Ukraine und Israel wiesen laut WHO im Zeitraum vom 01.07.2018 bis 30.06.2019 die weltweit höchsten Inzidenzen für Masern auf. Masernausbrüche sind ein Zeichen unzureichender Impfquoten, die durch vielfältige strukturelle und psychologische Barrieren verursacht werden. Strukturelle Barrieren für Masernimpfungen, wie mangelnde Routineimpfprogramme, bestehen vor allem in fragilen Ländern u. a. durch bewaffnete Konflikte. Sie wurden jedoch auch in Subpopulationen einkommensstärkerer Länder als Hauptursachen für geringe Masernimpfquoten u. a. durch fehlende Ressourcen für Impfdienste identifiziert. Psychologische Barrieren und nachfolgende Impfskepsis waren hauptsächlich in entwickelten Ländern mit gut funktionierenden Gesundheitssystemen und hohem Lebensstandard verbreitet.

**Diskussion:**

Die Gründe für die globale Masernkrise sind vielfältig und existieren teilweise schon seit Jahrzehnten. Sie scheinen sich jedoch inzwischen zu akkumulieren und seit 2018 dramatisch auf die Fallzahlen auszuwirken. Das Ziel, die Masern zu eliminieren, und die Aufrechterhaltung der hierfür notwendigen Impfprogramme sind ständige Herausforderungen, welche die strikte und permanente Einhaltung der WHO-Empfehlungen erfordern. Auch in Deutschland liegt die Anzahl der übermittelten Masernfälle immer noch auf einem Niveau deutlich über dem im Nationalen Impfplan festgelegten Leitziel zur Eliminierung der Masern. Immer wieder kommt es zu zeitlich begrenzten regionalen bis bundesweiten Ausbrüchen. Da Infektionserreger grenzübergreifend übertragen werden können, ist die internationale Perspektive ein wesentlicher Bestandteil der nationalen Gesundheitspolitik in Deutschland.

## Abstract (en)

**Background and aim:**

Despite a safe and effective vaccine being available for many years, the number of measles cases has been increasing again worldwide since 2018. Our report aims to identify putative reasons for this development.

**Methods:**

We conducted a selective literature search. Further, current reports and data from the World Health Organization (WHO), the United Nations Children’s Fund (UNICEF), and the World Bank were evaluated.

**Results:**

According to the WHO, Madagascar, the Ukraine, and Israel had the highest incidences of measles worldwide between 1 July 2018 and 30 June 2019. Measles outbreaks are a sign of inadequate vaccination rates caused by multiple structural and psychological barriers. Structural barriers to measles vaccination, such as a lack of routine vaccination programs, have been identified as the main cause of low measles vaccination rates, particularly in fragile countries e.g. due to armed conflicts, but also in some subpopulations of higher-income countries e.g. due to lacking resources for vaccination services. Psychological barriers leading to vaccination skepticism were prevalent mainly in developed countries with well-functioning health systems and a high standard of living.

**Conclusion:**

The reasons for the global measles crisis are manifold and in some cases have existed for decades. However, the consequences appear to be accumulating and have had a dramatic impact on case numbers since 2018. The goal of measles elimination and maintenance of the necessary vaccination programs is a constant challenge that requires strict and permanent compliance with WHO recommendations. The number of measles cases reported in Germany is still at a level above the key target for measles elimination specified in the national immunization plan. Timely and/or locally restricted as well as nationwide outbreaks continue to occur. Since infectious agents can be transmitted across borders, the international perspective is an essential component of national health policy in Germany.

## Hintergrund

Das Kinderhilfswerk der Vereinten Nationen (UNICEF) und die Weltgesundheitsorganisation (WHO) weisen bereits seit 2018 auf die erneute globale Zunahme der Masernfälle hin [1, 2]. Für das Jahr 2019 wurde der WHO in der Region Afrika ein Anstieg der Fallzahlen um das Zehnfache, in Europa um das Doppelte und in der östlichen Mittelmeerregion um das Eineinhalbfache im Vergleich zum selben Zeitraum im Vorjahr 2018 gemeldet (Stand: 31.07.2019). In den Regionen Südostasien und Amerika gab es einen leichten Rückgang der Masernfallzahlen um jeweils 15 % [2].

Die Masernerkrankung kann langwierig sein und komplikationsreich verlaufen. Sie führt zu einer transitorischen Schwächung des Immunsystems, die Monate anhalten kann [3]. Als mögliche Komplikationen dieser hochansteckenden Viruserkrankung können u. a. Lungenentzündung sowie eine fast immer tödlich verlaufende neurodegenerative Erkrankung des Gehirns, die subakute sklerosierende Panenzephalitis (SSPE), auftreten [4]. Die Letalität der Masern wird auf 1–3 pro 1000 Fälle geschätzt. Besonders Kinder unter 5 Jahren, Immungeschwächte, Schwangere, Personen mit Vitamin-A-Mangel und Erwachsene über 20 Jahre haben ein erhöhtes Risiko für schwere Erkrankungen, Komplikationen und Tod nach einer Maserninfektion [4].

Es existiert ein sicherer, effektiver und kostengünstiger Impfstoff. Darüber hinaus ist der Mensch der einzige Wirt für das Virus [5]. Deshalb ist es potenziell möglich, nach einer globalen Elimination in allen Regionen der WHO die Masern vollständig zu eradizieren. Die Elimination der Masern ist entsprechend der WHO-Definition erreicht, wenn die Mitgliedsstaaten eine Unterbrechung der endemischen Übertragung für mindestens 36 Monate mithilfe eines qualitativ hochwertigen Surveillance-Systems nachweisen können [6]. Hierfür müssen mindestens 95 % der Bevölkerung eine Immunität gegen die Masern besitzen, um durch die Ausbildung eines Gemeinschaftsschutzes die weitere Übertragung zu verhindern [7]. Der Global Vaccine Action Plan (GVAP) der WHO hatte das Ziel, Masern in mindestens 5 der 6 WHO-Regionen bis zum Jahr 2020 zu eliminieren [8, 9]. Die globale Impfquote der 1. Masernimpfdosis stagniert derzeit jedoch bei 86 % und keine der 6 WHO-Regionen konnte die Masernelimination erreichen oder aufrechterhalten [9].

Mögliche Gründe für die globale Zunahme der Fallzahlen, einschließlich struktureller und psychologischer Barrieren der Masernbekämpfung, sollen in diesem Bericht beispielhaft identifiziert und erläutert werden (Schlüsselaussagen siehe Infobox 1). Da Infektionserreger grenzübergreifend übertragen werden können, ist die internationale Perspektive ein wesentlicher Bestandteil der nationalen Gesundheitspolitik in Deutschland. Weiterführende Informationen zu deutschen Maserninfektionszahlen können den jüngsten Berichten (2018/2019) der Nationalen Verifizierungskommission Masern/Röteln entnommen werden [10, 11].

## Methoden

Es wurden eine selektive Literaturrecherche sowie eine Auswertung der aktuellen Veröffentlichungen bzw. länderspezifischen Daten der WHO, UNICEF und der Weltbank zu Masern, Impfquoten und Einkommenskategorien durchgeführt (Datenstand: 31.08.2019). Die Inzidenz der Masern (Fälle pro 1.000.000 Einwohner) ist jeweils für den Zeitraum 01.07.2018–30.06.2019 angegeben. Die durch UNICEF ermittelten Impfquoten sowie die von der Weltbank definierten Einkommenskategorien sind für das Jahr 2018 angegeben. Die psychologischen Barrieren wurden mithilfe des 5C-Modells nach Betsch näher erläutert [12].

## Ergebnisse

### Die aktuelle globale Epidemiologie der Masern

**Figure 1:**
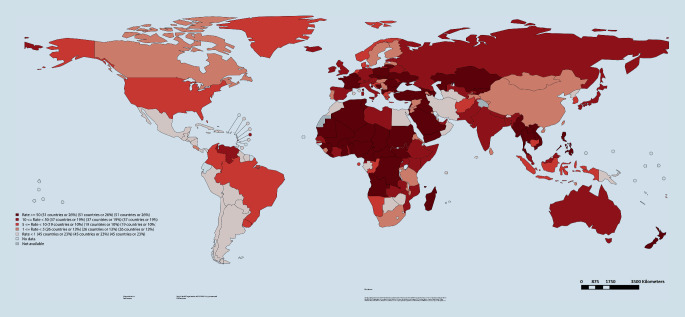

Eine Übersicht der Masernfälle und Inzidenzraten für den Zeitraum 01.07.2018 bis 30.06.2019 sowie zu Impfquoten für die erste (MCV-1) und zweite Impfdosis (MCV-2) für alle in diesem Situationsbericht erwähnten Länder sind in Abb. 1 bzw. Tab. 1 dargestellt [13]. In diesem Zeitraum wies Madagaskar mit 6065 Fällen pro 1.000.000 Einwohner die höchste Inzidenz auf. Die Impfquote für MCV‑1 lag hier bei 62 %. Angaben zur zweiten Impfung lagen nicht vor. In Europa hatte die Ukraine die höchste Inzidenz, wobei hier die Impfquoten bei 91 % (MCV-1) bzw. 90 % (MCV-2) lagen.

**Table 1:** 

Land	Pro-Kopf-Einkommen (laut Weltbank)	WHO-Region	Inzidenzrate (Fälle/1.000.000 Einwohner)	Fallzahlen	Impfquote (%) MCV‑1 (2018)	Impfquote (%) MCV‑2 (2018)
Madagaskar	Geringes Einkommen	Afrika	6064,6	150.976	62	k. A.
Ukraine	Mittleres Einkommen im unteren Bereich	Europa	1917,6	84.394	91	90
Israel	Hohes Einkommen	Europa	471,1	3982	98	96
Philippinen	Mittleres Einkommen im unteren Bereich	Westpazifik	443,7	45.847	67	40
Jemen	Mittleres Einkommen im unteren Bereich	Östliches Mittelmeer	435,1	12.001	64	46
Venezuela	Mittleres Einkommen im oberen Bereich	Amerika	177,3	6000	74	39
Bulgarien	Mittleres Einkommen im oberen Bereich	Europa	147,7	1039	93	87
Nigeria	Mittleres Einkommen im unteren Bereich	Afrika	138,8	25.814	65	k. A.
DR Kongo^a^	Geringes Einkommen	Afrika	117,4	9244	80	k. A.
Thailand	Mittleres Einkommen im oberen Bereich	Südostasien	108,3	7346	96	87
Rumänien	Mittleres Einkommen im oberen Bereich	Europa	83,1	1628	90	81
Brasilien	Mittleres Einkommen im oberen Bereich	Amerika	49,3	10.241	84	69
Serbien	Mittleres Einkommen im oberen Bereich	Europa	15,6	137	92	90
Vereinigtes Königreich	Hohes Einkommen	Europa	10,5	699	92	88
USA	Hohes Einkommen	Amerika	4,6	1536	94	92
Deutschland (im Vergleich)	Hohes Einkommen	Europa	6,8	559	97	93

k. A. keine Angabe

^a^*DR* Demokratische Republik

Für DR Kongo wurden für 2018 insgesamt 67.072, für 2019 sogar 140.725 aggregierte Fälle gemeldet, sodass die tatsächliche Inzidenzrate wahrscheinlich sehr viel höher liegt (1675,3 Fälle/1.000.000 Einwohner)

Quellen: Impfdosis (UNICEF; [22]), Fallzahlen/Inzidenzen (WHO; [13] oder WHO EURO [44]), Pro-Kopf-Bruttonational-Einkommen (nach Weltbank; [45])

### Strukturelle Barrieren bei der Masernbekämpfung

Hauptursachen für den globalen Anstieg der Masernfallzahlen sind laut UNICEF Konflikte und Instabilität sowie schwache Gesundheitssysteme [1]. Diese können zu strukturellen Barrieren der Masernbekämpfung führen, die in Tab. 2 zusammengefasst sind [14]. Nicht selten treten mehrere dieser Barrieren gleichzeitig auf. Der Zugang zu medizinischen Dienstleistungen einschließlich Impfangeboten kann dadurch kurz- oder langfristig in Ländern oder Landesteilen für die gesamte Bevölkerung oder Subpopulationen erschwert bzw. verhindert sein.

**Table 2:** 

Strukturelle Barrieren	Ursachen	Beispielländer	Betroffene Bevölkerungsgruppen
Fehlende Infrastruktur (Stromausfälle, fehlende Wasser- und Abwasserversorgung, unterbrochene Kühlketten)	z. B. Naturkatastrophen (Hurrikans), geografische Isolation, politische Unsicherheiten, Migration, Wirtschaftskrisen	DR Kongo, Jemen, Madagaskar, Nigeria, Philippinen	Gesamte Bevölkerung, Subpopulationen in schwer zugänglichen Gebieten
Fehlen/Unterbrechung/Beendigung von Routineimpfprogrammen	z. B. Naturkatastrophen (Hurrikans), politische Unsicherheiten, Krieg und Bürgerkrieg, Wirtschaftskrisen, Ausbrüche anderer Infektionskrankheiten, fehlende Mittel zur Aufrechterhaltung der Kontrollprogramme	DR Kongo, Jemen, Madagaskar, Nigeria, Ukraine, Venezuela	Gesamte Bevölkerung, Subpopulationen in bestimmten Regionen
Impfstoffmangel	z. B. Naturkatastrophen (Hurrikans), geografische Isolation, politische Unsicherheiten, Migration, Wirtschaftskrisen, mangelndes politisches Interesse	DR Kongo, Jemen, Madagaskar, Nigeria, Ukraine, Rumänien, Venezuela	Gesamte Bevölkerung, Subpopulationen in schwer zugänglichen Gebieten
Nicht zumutbare Reisedistanzen zu Impfzentren	z. B. geografische Isolation, Migration	DR Kongo, Madagaskar, Nigeria, Philippinen Ukraine, Thailand, Venezuela	Subpopulationen in schwer zugänglichen/ländlichen Gebieten
Schlechte Kommunikation von Impfprogrammen	z. B. mangelndes politisches Interesse, schwache Gesundheitssysteme einschließlich fehlenden Gesundheitspersonals, Wirtschaftskrisen	Philippinen, Thailand	Gesamte Bevölkerung, bestimmte Subpopulationen (z. B. bildungsferne Populationen ländlicher Gebiete)
Schlechter Zugang zum Gesundheitssystem einschließlich Impfangebote, Nichterfassung durch vorhandene staatliche Routineprogramme	z. B. geografische/soziale Isolation, politische Unsicherheiten, Migration	Brasilien, Bulgarien, DR Kongo, Jemen, Madagaskar, Nigeria, Rumänien, Serbien, Thailand, Ukraine, Venezuela	Gesamte Bevölkerung, Subpopulationen in schwer zugänglichen/ländlichen Gebieten, ethnische Minderheiten, indigene Populationen (Brasilien, Venezuela)
Schwache Surveillance-Systeme (für Masernfälle und Impfung)	z. B. schwache Gesundheitssysteme einschließlich fehlenden Gesundheitspersonals, Wirtschaftskrisen	DR Kongo, Madagaskar, Nigeria, Philippinen, Ukraine	Gesamte Bevölkerung, Subpopulationen in bestimmten Regionen
Verspätete/ungeeignete Maßnahmen bei Masernausbrüchen	z. B. schwache Gesundheitssysteme einschließlich fehlenden Gesundheitspersonals, Wirtschaftskrisen	DR Kongo, Philippinen, Rumänien, Ukraine, Venezuela	Gesamte Bevölkerung, Subpopulationen in bestimmten Regionen

#### Beispiele für strukturelle Barrieren in der gesamten Bevölkerung

- Die Ausbruchsbekämpfung in *Madagaskar* wurde laut WHO durch bewaffnete Konflikte nach den Wahlen im Dezember 2018, die geografische Isolation einiger Fälle, politische Unsicherheit sowie Naturkatastrophen (Hurrikans) erschwert. Durch saisonal auftretende Ausbrüche der Pest waren die Gesundheitsbehörden in Madagaskar zusätzlich belastet [15].
- In der *Demokratischen Republik Kongo* (DR Kongo) stellen die instabile Sicherheitslage, die schwere geografische Zugänglichkeit einiger Provinzen und intensive Bevölkerungsbewegungen sowie der noch andauernde Ebolaausbruch im Osten des Landes, der Ressourcen bindet und Unsicherheiten in der Bevölkerung verstärkt, eine enorme Herausforderung auch bei der Bekämpfung der Masern dar [16].
- Im Nordosten *Nigerias* gibt es seit 2009 eine humanitäre Krise (ausgelöst durch terroristische Aktivitäten der „Boko Haram“) mit mehr als 7 Mio. betroffenen und hilfsbedürftigen Menschen in den Bundesstaaten Borno, Adamwa und Yobe. Infolgedessen sind seit Beginn des Konflikts 1,8 Mio. Menschen auf der Flucht und suchen in Flüchtlingslagern Schutz, wo das Übertragungsrisiko von Infektionskrankheiten, insbesondere Masern, sehr hoch ist [17].
- *Jemen*, eines der ärmsten Länder im Mittleren Osten, ist seit 2010 Schauplatz zahlreicher bewaffneter Konflikte, die verheerende Auswirkungen auf die lokale Bevölkerung haben. Stromausfälle, fehlende Wasser- und Abwasserversorgung sowie unterbrochene Kühlketten behindern die Gesundheitsversorgung einschließlich der Versorgung mit Impfangeboten für die Bevölkerung massiv [18].
- Auch für die *Philippinen*, wo das nationale Gesundheitsministerium im Februar 2019 einen landesweiten Masernausbruch erklärte, nennt die WHO nichtfunktionierende Kühlketten für Impfstoffe als großes Problem bei der Ausbruchsbekämpfung. Darüber hinaus fehlt ein Kontrollsystem für die lückenlose Durchimpfung von Zielgruppen. Es mangelt an detaillierten Analysen, um gezielte Interventionen für die stark betroffenen Gebiete zu identifizieren. Informationsmaterial über Masern und Masernimpfungen, welches insbesondere Ängste im Zusammenhang mit Impfstoffen adressieren soll, erreicht die Bevölkerung nicht ausreichend [19].
- In der *Ukraine* nennt die WHO Herausforderungen bei der Bereitstellung, Lagerung und Handhabung des Impfstoffs als massive Barrieren bei der Ausbruchsbekämpfung. Das Gesundheitssystem des Landes ist durch die militärischen Konflikte infolge der Annexion der Krim, politische Instabilität und eine desolate Wirtschaftslage stark belastet. Infolgedessen wurden Routineimpfungen nicht oder nur noch unzureichend durchgeführt. Die Impfquoten lagen seit den 1980er-Jahren bis 2008 konstant bei mindestens 94 %. Von 2009 bis 2016 sanken diese jedoch auf 42 % für die erste und 31 % für die 2. Impfdosis, was einem der niedrigsten Niveaus weltweit entspricht [20]. Weitere Gründe für diese dramatische Entwicklung sind laut WHO neben den bereits genannten strukturellen Barrieren auch ein geringes Vertrauen des Gesundheitspersonals in die Impfung sowie eine geringe Nachfrage durch die Bevölkerung. Auf diese Faktoren wird im Abschnitt „Psychologische Barrieren“ näher eingegangen.

Humanitäre Krisen und Instabilität können auch Länder mit höherem Pro-Kopf-Einkommen betreffen.

- *Venezuela* befindet sich seit 2010 in einer andauernden schweren sozioökonomischen und politischen Krise, in deren Folge das Gesundheitssystem vielerorts zusammenbrach. So betrug die Inflationsrate im Jahr 2016 rund 260 % und stieg 2018 auf über 65.000 %. Qualifiziertes Fachpersonal einschließlich biomedizinischer Forscher und Gesundheitspersonal verlassen das Land. Mehr als 280.000 Kinder sind durch schwere Unterernährung gefährdet [21]. Das erneute Auftreten der Masern in Venezuela seit 2017 ist eine Folge des unterbrochenen nationalen Immunisierungsprogramms, wodurch Routineimpfungen nicht mehr überall stattfanden. Die Impfquote für die 2. Impfdosis betrug 2018 laut UNICEF nur noch 39 % [22].
- Die ehemals sehr hohen Impfquoten der Kinder in *Rumänien* von mindestens 95 % zu Beginn der 2000er-Jahre konnten in den vergangenen Jahren nicht mehr aufrechterhalten werden und sanken 2017 auf einen Tiefpunkt von 75 % für die 2. Impfdosis [20]. Als Gründe werden vom WHO-Regionalbüro für Europa (WHO EURO) Veränderungen des Gesundheitssystems, Knappheit des Impfstoffs sowie die Komplexität eines adäquaten Ausbruchsmanagements genannt.

#### Beispiele für strukturelle Barrieren in Subpopulationen

Die aktuellen Masernimpfquoten in der europäischen Region waren nach Angaben der WHO EURO mit 95 % für die 1. Impfdosis und 90 % für die 2. Impfdosis nie besser [20]. Trotzdem kam es im Jahr 2018 zu einem dramatischen Anstieg der Masernfälle in Europa auf ein Rekordniveau. Die WHO EURO begründet dies mit einer steigenden Anzahl von Clustern nichtimmunisierter Bevölkerungsgruppen in der europäischen WHO-Region [20]. Der Zugang zu Impfangeboten kann für einige Subpopulationen auch in Ländern mit ansonsten gutem Zugang zu medizinischen Dienstleistungen schwierig sein. Hohe durchschnittliche Impfquoten auf nationaler Ebene können dazu führen, bestehende niedrige Impfquoten auf lokaler Ebene zu überschätzen bzw. nicht wahrzunehmen.

- Eine 2012 veröffentlichte Studie von 468 Romakindern in *Serbien* zeigte, dass nur 14 % der untersuchten Kinder einen vollständigen, altersgerechten Impfschutz gegen Masern hatten [23]. Die durchschnittliche Masernimpfquote lag in Serbien zu dieser Zeit bei 90 % [22]. Hauptrisikofaktor für einen unzureichenden Masernimpfstatus in dieser Gruppe war das Fehlen einer Registrierung durch die staatlichen Behörden. Die Kinder waren im öffentlichen Gesundheitssystem nicht erfasst, sodass sie durch staatliche Routineimpfprogramme nicht erreicht wurden.
- Für eine jüdisch-orthodoxe Gemeinschaft in London (*Vereinigtes Königreich*) wurde herausgefunden, dass nicht kulturelle und religiöse Ablehnung von Impfungen der Grund für geringe Impfquoten in dieser Gruppe war. Vielmehr standen die öffentlichen Gesundheitsdienste nach der Identifizierung der Impflücken in dieser Gruppe vor der Herausforderung, einer großen Anzahl von Kindern ohne zusätzliche Ressourcen Immunisierungsdienste anzubieten [24].

**Weitere Beispiele anderer Regionen**
- Der aktuelle Masernausbruch in *Thailand* betrifft vor allem die arme Bevölkerung schwer zugänglicher Gebiete in den südlichen Provinzen. Die meisten Betroffenen sind nichtgeimpfte Kinder aus armen und bildungsfernen Familien oder Kinder eingewanderter Arbeiter, die keine Kenntnis von dem Ausbruch und einen schlechten Zugang zu öffentlichen Gesundheitsdienstleistungen haben [19].
- In *Venezuela* und *Brasilien* sind indigene Populationen sehr stark durch die aktuellen Masernausbrüche betroffen. Infolge der wirtschaftlichen Krise etablierten sich illegale Bergbaucamps in den indigenen Gebieten Venezuelas. Diese ziehen Arbeiter aus anderen Regionen Venezuelas, Nachbarländern und indigenen Siedlungen an. Maserntransmissionen sind innerhalb der Camps und darüber hinaus bei Rückkehr in die Heimatregionen und indigenen Siedlungen möglich [21]. Humanitäre Hilfe, einschließlich Impfungen, ist in dieser vulnerablen Population aufgrund der Abgeschiedenheit der Siedlungsgebiete und der seminomadischen Lebensweise nur begrenzt möglich [21].

#### Zusammenfassung der strukturellen Barrieren

Strukturelle Barrieren sind vor allem in fragilen Ländern wie Madagaskar, der DR Kongo und Jemen die Hauptursache für geringe Masernimpfquoten und hohe Masernfallzahlen in der gesamten Bevölkerung. Sie spielen allerdings auch in einigen Subpopulationen einkommensstärkerer Länder mit sehr guten Routineimpfprogrammen (z. B. Romakinder in Serbien) eine Rolle.

Für eine erfolgreiche Elimination der Masern und deren Aufrechterhaltung müssen die WHO-Empfehlungen einschließlich Surveillance, schnellen Ausbruchsmanagements, Routineimpfungen und zusätzlicher Impfungen bestimmter Zielgruppen strikt eingehalten werden. Länder, die diese Maßnahmen nicht aus eigener Kraft umsetzen können, benötigen hierbei die Unterstützung der Weltgemeinschaft sowie von nationalen und internationalen Organisationen.

### Psychologische Barrieren der Masernbekämpfung

Masernausbrüche sind ein Zeichen unzureichender Impfquoten. Die Strategic Advisory Group of Experts (SAGE) der WHO definiert den Begriff Impfskepsis (Vaccine Hesitancy) als die Verzögerung von Impfungen oder Ablehnung von Impfstoffen, obwohl sie verfügbar wären [25]. Die Impfskepsis zählt laut WHO zu einer der 10 größten Bedrohungen der globalen Gesundheit [26]. Sie tritt sehr heterogen und kontextspezifisch auf und variiert je nach Zeit, Ort und Impfstoff [25]. SAGE und verschiedene Forschungsgruppen haben Modelle entwickelt, um psychologische Gründe des Nichtimpfens zu analysieren [12, 27]. Eines dieser Modelle, das „5C-Modell“, erklärt die Impfentscheidung anhand der folgenden 5 Determinanten: Confidence (Vertrauen), Complacency (Risikowahrnehmung), Constraints/Convenience (Barrieren in der Ausführung), Calculation (Ausmaß der Informationssuche) und Collective Responsibility (Verantwortungsgefühl für die Gemeinschaft; [12]).

#### Confidence

Das Vertrauen in die Effektivität und Sicherheit von Impfungen, das Gesundheitssystem und die Motive der Entscheidungsträger, welche Impfempfehlungen geben, spielt eine wichtige Rolle bei der Impfentscheidung. Je höher das Vertrauen, desto wahrscheinlicher ist eine hohe Impfquote in der Bevölkerung. Eine in mehr als 140 Ländern durchgeführte Studie mit über 140.000 Teilnehmenden zur Wahrnehmung von Wissenschaft und Gesundheit in der Welt fand heraus, dass in Ländern mit einem hohen Pro-Kopf-Einkommen weniger Menschen glauben, dass Impfstoffe sicher seien. Dieser Aussage („Impfungen sind sicher“) stimmten in Nordeuropa nur 75 %, in Nordamerika nur 72 %, in Westeuropa nur 59 % und in Osteuropa sogar nur 50 % zu. In Regionen mit einem geringen Pro-Kopf-Einkommen ist das Vertrauen in Impfstoffe hingegen wesentlich höher. In Südasien hielten 95 % und in Ostafrika 92 % der Befragten Impfstoffe für sicher [28].

Missverständnisse und Fehlinformationen beeinflussen das Vertrauen in einen Impfstoff. 1998 erschien in der renommierten medizinischen Wissenschaftszeitschrift *Lancet* ein Artikel britischer Wissenschaftler, in dem Fälle von Autismus mit dem Mumps-Masern-Röteln(MMR)-Impfstoff in Verbindung gebracht wurden [29]. Obwohl dieser Artikel 2010 aufgrund wissenschaftlicher Mängel zurückgezogen und inhaltlich bereits mehrfach von wesentlich größeren und wissenschaftlich hochwertigeren Studien widerlegt wurde, führt diese Information bis heute zu Verunsicherungen und wird weiterhin von Impfgegnern als Argumentationsgrundlage genutzt [30, 31]. Ein weiterer Grund für die dramatische Entwicklung der Masern in der *Ukraine* ist laut WHO auch das geringe Vertrauen des Gesundheitspersonals in die Impfung [20]. Dies sei die Folge eines unterfinanzierten Gesundheitssystems, aber auch gezielter Desinformationskampagnen, welche das ukrainische Impfprogramm destabilisierten [32]. Auch für den aktuellen Masernausbruch in *Thailand*, der hauptsächlich Kinder betrifft, nannte das Regionalbüro der WHO für die westpazifische Region mangelndes Vertrauen der Eltern in den Masernimpfstoff als eine der Hauptursachen [19]. Das Vertrauen kann auch durch Schwierigkeiten mit anderen Impfstoffen, wie beispielsweise dem Dengueimpfstoff Dengvaxia auf den *Philippinen*, empfindlich gestört werden und wirkt sich auch auf die Akzeptanz der Masernimpfung aus.

#### Complacency

Complacency beschreibt das individuell wahrgenommene Krankheitsrisiko, d. h. inwieweit sich der Einzelne anfällig für bestimmte Infektionserkrankungen fühlt und Impfungen als notwendig ansieht. Complacency liegt vor, wenn das Risiko impfpräventabler Erkrankungen als gering eingeschätzt wird und Impfungen nicht als notwendige vorbeugende Maßnahme angesehen werden. Dies ist ein weiterer wichtiger Faktor der Entscheidung für oder gegen eine Impfung [27]. Eine fehlende Risikowahrnehmung für Masern ist paradoxerweise dem Erfolg des Masernimpfstoffs zuzuschreiben, wodurch die Erkrankung und damit assoziierte Komplikationen bei hohen Durchimpfungsraten kaum noch sichtbar sind. Dies ist vor allem in Ländern mit hohen Impfquoten, wie der WHO-EURO-Region, verbreitet, wurde jedoch auch als weitere Ursache des aktuellen Masernausbruchs in *Thailand* evident [19, 20].

#### Constraints (Convenience)

Constraints, oder auch Barrieren in der Ausführung, beschreiben das Ausmaß individuell wahrgenommener struktureller Hürden, die das Annehmen von Impfangeboten verhindern. Diese schließen Stress, den finanziellen und zeitlichen Aufwand oder Verständnisschwierigkeiten ein. Entscheidend ist auch, ob Impfen als wichtig genug angesehen wird, um diese Barrieren zu überwinden [27]. Constraints sind relevant, wenn beispielsweise negative Einflüsse in der physischen Verfügbarkeit, Erschwinglichkeit und Zahlungsbereitschaft, in geografischer Erreichbarkeit, Verständlichkeit (Sprach- und Gesundheitskompetenz) und Anziehungskraft des Impfprogramms die Impfungen beeinflussen [27]. Diese Determinante ist eng mit strukturellen Barrieren, welche einer ganzen Bevölkerungsgruppe oder bestimmten Subpopulationen den Zugang zu Gesundheitsleistungen und Impfangeboten erschweren, verbunden (→ Strukturelle Barrieren).

Für die Romapopulation in *Bulgarien* identifizierte die WHO EURO, dass die Qualität der Begegnung mit dem Gesundheits- und Pflegepersonal der entscheidende Faktor für die Akzeptanz von Impfangeboten in dieser Bevölkerungsgruppe war. Es mangelte also an einer Versorgung, die auch Roma willkommen hieß und vorbehaltslos versorgte [25]. Trotz ergriffener Maßnahmen einschließlich des Einsatzes sogenannter Roma Health Mediators traten jedoch weiterhin überdurchschnittlich viele Masernfälle in der bulgarischen Romapopulation auf [33–35].

Zwischen März 2018 und Juni 2019 kam es in *Israel* zu einem Masernausbruch mit mehr als 4000 Fällen. Etwa die Hälfte der Fälle (*n* = 2202) traten in Jerusalem, hauptsächlich bei nichtgeimpften Kindern jüdisch-orthodoxer Gemeinschaften, auf. Kinder in diesen orthodoxen Gemeinschaften Jerusalems wiesen mit nur 78 % eine geringe Impfquote für die erste Dosis der Masernimpfung im Vergleich zu nationalen Impfquoten (98 % für MCV‑1, Tab. 1) auf [36]. Die sich anschließenden Maßnahmen (12-Stunden-Schichten in Mutter-Kind-Kliniken ausschließlich für Impfungen, Gesundheitsdienste in Schulen, mobile Impfeinheiten in den orthodoxen Stadtvierteln Jerusalems) machten Impfungen für die betroffene Subpopulation leicht zugänglich. So konnte die Impfquote für die erste Masernimpfung in allen Mutter-Kind-Kliniken der betroffenen Stadtviertel im Anschluss an diese und weitere Maßnahmen auf 96 % gesteigert werden [36].

#### Calculation

Der Begriff Calculation drückt den individuellen Grad der aktiven Informationssuche von Personen aus. Personen, die sich über Masernimpfungen informieren wollen, stehen häufig vor der schwierigen Aufgabe, seriöse von nichtseriösen Informationsangeboten (Internetseiten, Flyer, Broschüren) zu unterscheiden. Fehlinformationen sind häufig schwer von seriösen Informationen zu unterscheiden, da viele impfkritische Internetangebote ein professionelles Erscheinungsbild haben und Wissenschaftlichkeit suggerieren [27]. Personen mit hohen Calculation-Werten haben häufig sogar mehr Falschwissen und eine geringere Impfbereitschaft, da sie bei ihrer Recherche auf Fehlinformationen und Verschwörungstheorien von Impfgegnern stoßen. Studien zeigen, dass bereits ein kurzer Besuch auf impfkritischen Internetseiten zu Verunsicherungen führt und das Impfverhalten negativ beeinflussen kann [37].

Masern galten in den *USA* bereits im Jahr 2000 als eliminiert [38], jedoch treten immer wieder Ausbrüche wie derzeit in Kalifornien, New York, Pennsylvania und Washington auf [39]. Das US-amerikanische Center for Disease Control and Prevention sieht das größte Risiko bei nichtgeimpften Reisenden, die sich im Ausland mit Masern infizieren und anschließend in Gemeinschaften mit geringen Impfquoten zurückkehren [40]. So geht der aktuelle Ausbruch in New York auf nichtgeimpfte Reiserückkehrer aus *Israel* und die anschließende Übertragung auf Mitglieder ihrer jüdisch-orthodoxen Gemeinde mit geringen Impfquoten zurück [41]. Ein Beispiel für sehr aufwendig aufbereitete Fehlinformationen zu Impfungen ist das durch Impfgegner innerhalb der jüdisch-orthodoxen Gemeinschaft in den USA verbreitete Handbuch „Parents educating and advocating for Children’s Health“ (Peach). Dies verstärkt die Impfskepsis in dieser Bevölkerungsgruppe [42].

### Collective Responsibility

Der Masernimpfstoff ist ein Lebendimpfstoff und ab einem Lebensalter von mindestens 9 Monaten zugelassen. Er ist für Schwangere und Personen mit bestimmten Immundefizienzen kontraindiziert. Diese vulnerablen Bevölkerungsgruppen, in denen Komplikationen und Todesfälle nach einer Maserninfektion besonders häufig auftreten, sind daher auf den Gemeinschaftsschutz angewiesen [6]. Hohe Collective-Responsibility-Werte gehen mit höherer Empathie und dem Zugehörigkeitsgefühl zu einer Gruppe einher [27]. Ein Ziel des Global Vaccine Action Plan der WHO (GVAP Strategic Objective 2) sieht vor, dass Individuen und Gesellschaften/Gemeinschaften den Wert von Impfungen anerkennen und Impfungen sowohl als ihr Recht als auch ihre Pflicht einfordern [25]. Interessanterweise wird diese Determinante sowohl von Befürwortern als auch Gegnern der Masernimpfung genutzt. Jüdische Rabbiner in den *USA* berufen sich in ihrer Argumentation für die Masernimpfung auf religiöse Schriften, die den Schutz der eigenen Gesundheit und der Gesundheit anderer als hohe jüdische Werte ansehen. Im Gegensatz dazu adressieren auch Impfgegner wie die bereits erwähnte „Peach“-Anhängerschaft das Verantwortungsgefühl für die Gemeinschaft. In ihrem Handbuch behaupten sie, es gäbe keine größere Bedrohung der öffentlichen Gesundheit als Impfungen [42].

### Zusammenfassung der psychologischen Barrieren

Psychologische Barrieren wie mangelndes Vertrauen und fehlende Risikowahrnehmung sind in vielen Ländern eine wichtige Ursache für den Anstieg der Masernfallzahlen. Dies betrifft insbesondere Länder mit sehr gut funktionierenden Gesundheitssystemen und einem hohen Lebensstandard, ist aber auch in Ländern mit einem mittleren oder niedrigen Pro-Kopf-Einkommen (z. B. Philippinen, DR Kongo) zu beobachten. Gezielte Analysen sind notwendig, um herauszufinden, welche Gruppen (Altersgruppen, ethnische Minderheiten, bestimmte Einkommensgruppen, bestimmte Siedlungsgebiete etc.) Impflücken aufweisen. Darüber hinaus müssen die Barrieren, welche diese Bevölkerungsgruppen von einem ausreichenden Masernimpfschutz abhalten, identifiziert und ihre Bedarfe genau analysiert werden. Nur so können „maßgeschneiderte“ sinnvolle Interventionen für alle Gruppen mit Impflücken entwickelt, umgesetzt und evaluiert werden.

## Diskussion

Der weltweite besorgniserregende Anstieg der Masernfallzahlen hat vielfältige Ursachen und scheint sich ohne geeignete Gegenmaßnahmen weiter fortzusetzen.

Das Ziel einer dauerhaften weltweiten Masernelimination bleibt selbst in einkommensstarken Ländern mit sehr guten Routineimpfprogrammen, wie Südkorea, das Vereinigte Königreich und den USA, durch Veränderungen in der Impf-Compliance, anhaltende Transmissionen in benachbarten Regionen sowie geringe Impfraten in einigen Subpopulationen eine ständige Herausforderung. Der Status der erfolgreichen Masernelimination ist fragil. Dies wurde erst kürzlich deutlich, als 4 europäische Länder (*Albanien, Griechenland, Vereinigtes Königreich* und *Tschechien*) diesen Status erstmals seit Beginn des Verifizierungsprozesses in der WHO-EURO-Region im Jahr 2012 wieder verloren haben [43].

### Limitierende Faktoren der Daten

Dieser Bericht beruht auf einer selektiven Literaturrecherche und hat infolgedessen keinen Anspruch auf Vollständigkeit. Die verwendeten epidemiologischen Daten und Berichte der WHO unterliegen der Unsicherheit dieser Daten und können Abweichungen zu den tatsächlichen Fallzahlen aufweisen.

### Fazit

Die Gründe für den erneuten globalen Anstieg der Masernfallzahlen sind vielfältig. Masernausbrüche sind ein Resultat zu geringer Masernimpfquoten in einigen Subpopulationen oder in der gesamten Bevölkerung, die u. a. durch strukturelle und psychologische Barrieren entstehen können. Unbeantwortet bleibt die Frage, warum gerade jetzt die Zahl der Masernfälle weltweit ansteigt. Viele der hier genannten Barrieren und deren Ursachen existieren bereits seit mehreren Jahren und sogar Jahrzehnten (z. B. politische Instabilität in Venezuela, bewaffnete Konflikte in der Ukraine, humanitäre Krisen in der DR Kongo, Nigeria und Jemen, Impfskepsis in bestimmten Bevölkerungsgruppen). Die Folgen der über einen längeren Zeitraum vernachlässigten Masernbekämpfung sind z. B. die Anhäufung mehrerer nichtgeimpfter Jahrgänge und die Akkumulation ungeschützter Personengruppen, in denen sich die hochansteckenden Masern schnell ausbreiten können. Dies führt zu hohen Fallzahlen.

Es ist unerlässlich, die geschilderten Faktoren der Masernbekämpfung kontinuierlich zu bewerten und geeignete Maßnahmen zu entwickeln und umzusetzen, um ausreichende Impfquoten in allen Bevölkerungsgruppen zu erzielen und aufrechtzuerhalten. Ziel ist die Erreichung der Elimination und eines Tages auch der weltweiten Eradikation der Masern. Auch in Deutschland liegt die Anzahl der übermittelten Masernfälle auf einem Niveau deutlich über dem im Nationalen Impfplan festgelegten Leitziel zur Eliminierung der Masern. Da Infektionserreger grenzübergreifend übertragen werden können, sollte die internationale Perspektive auch ein wesentlicher Bestandteil der nationalen Gesundheitspolitik sein. Sonst bleibt auch in Ländern mit einer verifizierten Elimination der Masern das Risiko permanent bestehen, Masernfälle zu importieren, die wiederum zu Ausbrüchen führen können.

#### Infobox 1 Schlüsselaussagen des Beitrags

- Es ist seit 2018 eine erneute globale Zunahme von Masernfallzahlen zu beobachten.
- Die Gründe für die globale Masernkrise sind vielfältig und existieren teilweise schon seit Jahrzehnten.
- Masernausbrüche sind ein Zeichen unzureichender Impfquoten, die durch vielfältige strukturelle und psychologische Barrieren verursacht werden.
- Strukturelle Barrieren für Masernimpfungen, wie z. B. mangelnde Routineimpfprogramme verursacht durch bewaffnete Konflikte oder die eingeschränkte Zugänglichkeit von Subpopulationen zu Impfungen, wurden als Hauptursachen für geringe Masernimpfquoten in den Ländern identifiziert.
